# Reticulocyte Antioxidant Enzymes mRNA Levels versus Reticulocyte Maturity Indices in Hereditary Spherocytosis, β-Thalassemia and Sickle Cell Disease

**DOI:** 10.3390/ijms25042159

**Published:** 2024-02-10

**Authors:** Daniela Melo, Fátima Ferreira, Maria José Teles, Graça Porto, Susana Coimbra, Susana Rocha, Alice Santos-Silva

**Affiliations:** 1 UCIBIO—Applied Molecular Biosciences Unit, Laboratory of Biochemistry, Department of Biological Sciences, Faculty of Pharmacy, University of Porto, 4051-401 Porto, Portugal; danielamelo94@hotmail.com (D.M.); ssn.coimbra@gmail.com (S.C.); 2 Associate Laboratory i4HB—Institute for Health and Bioeconomy, Faculty of Pharmacy, University of Porto, 4051-401 Porto, Portugal; 3 Hematology Service, Centro Hospitalar e Universitário de São João, 4051-401 Porto, Portugal; marsoufer@hotmail.com; 4 Clinical Pathology, Centro Hospitalar e Universitário de São João, 4051-401 Porto, Portugal; mizeteles@hotmail.com; 5 Imuno-Hemotherapy Service, Centro Hospitalar Universitário de Santo António, 4051-401 Porto, Portugal; gporto@ibmc.up.pt; 6 Center for Predictive and Preventive Genetics (CGPP)/Institute for Molecular and Cellular Biology (IBMC), 4051-401 Porto, Portugal; 7 Abel Salazar Institute of Biomedical Sciences (ICBAS), University of Porto, 4051-401 Porto, Portugal; 8 1H-TOXRUN—One Health Toxicology Research Unit, University Institute of Health Sciences, CESPU, CRL, 4585-116 Gandra, Portugal

**Keywords:** reticulocyte, hereditary spherocytosis, β-thalassemia, sickle cell disease, antioxidant enzymes

## Abstract

The antioxidant enzymes superoxide dismutase (SOD), catalase (CAT), glutathione peroxidase (GPx) and peroxiredoxin 2 (Prx2) are particularly important in erythroid cells. Reticulocytes and other erythroid precursors may adapt their biosynthetic mechanisms to cell defects or to changes in the bone marrow environment. Our aim was to perform a comparative study of the mRNA levels of *CAT, GPX1, PRDX2* and *SOD1* in reticulocytes from healthy individuals and from patients with hereditary spherocytosis (HS), sickle cell disease (SCD) and β-thalassemia (β-thal), and to study the association between their transcript levels and the reticulocyte maturity indices. In controls, the enzyme mRNA levels were significantly correlated with reticulocyte maturity indices for all genes except for *SOD1*. HS, SCD and β-thal patients showed younger reticulocytes, with higher transcript levels of all enzymes, although with different patterns. β-thal and HS showed similar reticulocyte maturity, with different enzyme mRNA levels; SCD and HS, with different reticulocyte maturity, presented similar enzyme mRNA levels. Our data suggest that the transcript profile for these antioxidant enzymes is not entirely related to reticulocyte maturity; it appears to also reflect adaptive mechanisms to abnormal erythropoiesis and/or to altered erythropoietic environments, leading to reticulocytes with distinct antioxidant potential according to each anemia.

## 1. Introduction

Due to the increased premature destruction of red blood cells (RBCs) in congenital non-immune hemolytic anemias (NIHAs), reticulocytosis and the release of immature reticulocytes into the bloodstream are common features and reflect the erythropoietic response to anemia [1,2,3]. Reticulocyte analysis through the automated reticulocyte maturation indices is a valuable tool for assessing the erythropoietic status of NIHAs [4,5,6,7,8] and can be used for differential diagnosis [4,5,9,10,11,12,13,14,15]. In ineffective or stressed erythropoiesis, the reticulocyte count is usually lower than expected for anemia severity [6,15].

The reticulocytes (and other erythroid precursors) may adapt their biosynthetic mechanisms to cell defects or to an altered environment in the bone marrow, as occurs in NIHAs [16]. Contrary to mature RBCs, which contain no DNA or RNA, reticulocytes still have a network of ribosomal RNA [17].

In hereditary spherocytosis (HS), the membrane protein deficiencies induce membrane destabilization [1,18], triggering metabolic stress in the erythroid cell to maintain membrane integrity [19,20,21].

In β-thalassemia (β-thal), the tetramers of α-globin chains can precipitate and release heme and iron, creating an oxidative environment within erythroid cells [2,22].

In sickle cell disease (SCD), the high trend of hemoglobin (Hb) S to polymerize leads to cell sickling, inducing progressive cell dehydration and membrane damage by repeated sickling, creating metabolic stress within cells [3,23,24].

Superoxide dismutase (SOD), catalase (CAT), glutathione peroxidase (GPx) and peroxiredoxin 2 (Prx2) are important to assure a proper antioxidant environment within erythroid cells and, thereby, an adequate erythropoiesis and normal erythrocyte lifespan [25]. These antioxidant enzymes have been fairly characterized in mature RBCs; however, there are very few reports about their transcriptome profiles in reticulocytes from healthy individuals [26,27] and, especially, from patients with NIHAs [28,29].

Our aim was to better understand the modulation of the antioxidant enzymes in HS, SCD and β-thal patients and in healthy individuals by performing a comparative analysis of their reticulocyte maturation profiles and evaluating their correlation with the mRNA levels of *CAT, SOD1, GPX1* and *PRDX2.*

## 2. Results

### 2.1. Hematological Data

When compared to the control group, we found that RBC concentrations were significantly lower in HS and SCD patients and significantly higher in β-thal patients. This latter group also presented significantly lower mean corpuscular volume (MCV) and mean corpuscular Hb (MCH) (Table 1). Compared to the control group, Hb concentrations were significantly lower in all NIHAs, with SCD presenting the lowest value; all patients showed significantly higher red cell distribution width (RDW), and SCD patients showed the highest value (Table 1).

### 2.2. Reticulocyte Parameters

The reticulocytes (percentage and concentration) and the reticulocyte production index (RPI) were significantly increased in HS and SCD patients compared to the control group, with HS patients showing the highest values (Table 1). β-thal patients presented higher reticulocyte concentrations compared to controls, and their RPI was the lowest compared to all groups.

HS, SCD and β-thal patients presented significantly lower low-fluorescence reticulocytes (LFRs) and higher medium-fluorescence reticulocytes (MFRs), high-fluorescence reticulocytes (HFRs)and immature reticulocyte fraction (IRFs) compared to controls; SCD patients presented the lowest LFRs and the highest IRF (Table 1).

### 2.3. CAT, GPX1, PRDX2, SOD1 and GADPH mRNA Levels in Reticulocytes

**Figure 1:**
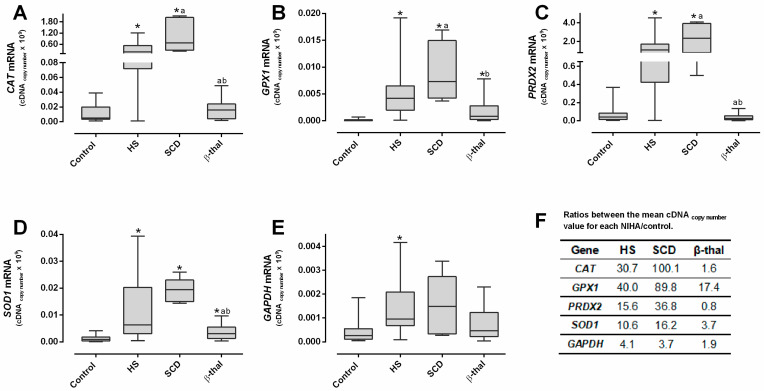
Reticulocyte mRNA levels of catalase (CAT, (**A**)), glutathione peroxidase 1 (GPX1, (**B**)), peroxiredoxin 2 (PRDX2, (**C**)), superoxide dismutase 1 (SOD1, (**D**)) and glyceraldehyde-3-phosphate dehydrogenase (GAPDH, (**E**)) in the control (*n* = 31), hereditary spherocytosis (*n* = 22), sickle cell disease (*n* = 6) and β-thalassemia (*n* = 20) groups. The embedded table (**F**) shows the comparison between the average mRNA transcript levels for each disease in relation to the control group (ratios). Data are presented as median (interquartile range) for (**A**–**E**). Mann–Whitney U test was used to compare differences between groups; *p* < 0.05 was considered statistically significant. * *p* < 0.05 vs. control group; ^a^ *p* < 0.05 vs. HS patients; ^b^ *p* < 0.05 vs. sickle cell disease patients. β-thal, β-thalassemia; HS, hereditary spherocytosis; SCD, sickle cell disease.

In HS and SCD patients, the reticulocytes showed significantly increased levels of mRNA of all the studied genes, compared to controls, while in β-thal patients, only *GPX1* and *SOD1* were significantly increased (Figure 1A–E). When comparing to HS or SCD, the β-thal reticulocytes presented significantly lower RNA levels of *CAT, PRXD2* and *SOD1* and significantly decreased transcripts of *GPX1* than SCD.

The cDNA copy-number values of all genes were several folds higher than controls in HS and SCD patients, with the latter presenting the highest values; in β-thal, only the *GPX1* and *SOD1* ratios were higher (Figure 1F).

### 2.4. Reticulocyte Maturity Indices versus mRNA Levels of CAT, GPX1, PRDX2, SOD1 and GAPDH 

**Figure 2:**
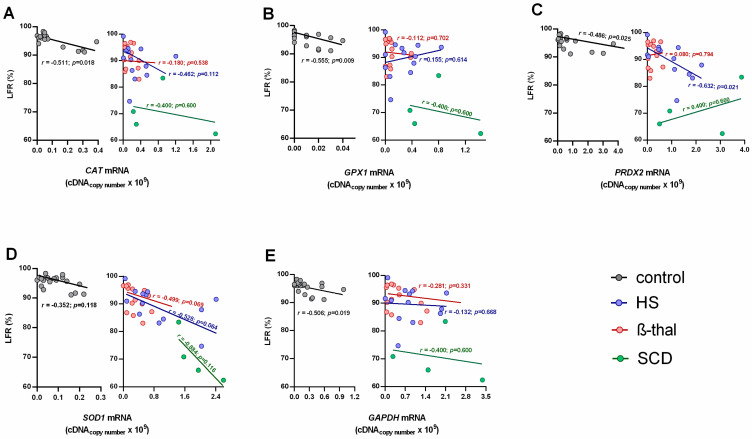
Percentage of low-fluorescence reticulocytes (LFR, %) versus mRNA levels of catalase (*CAT*, (**A**)), glutathione peroxidase 1 (*GPX1*, (**B**)), peroxiredoxin 2 (*PRDX2*, (**C**)), superoxide dismutase 1 (*SOD1*, (**D**)) and glyceraldehyde-3-phosphate dehydrogenase (*GAPDH*, (**E**)) for the control (*n* = 21), hereditary spherocytosis (*n* = 13), sickle cell disease (*n* = 4) and β-thalassemia (*n* = 14) groups. Spearman’s rank correlation coefficient was used to evaluate relationships between sets of data; *p* < 0.05 was considered statistically significant. β-thal, β-thalassemia; HS, hereditary spherocytosis; SCD, sickle cell disease.

**Figure 3:**
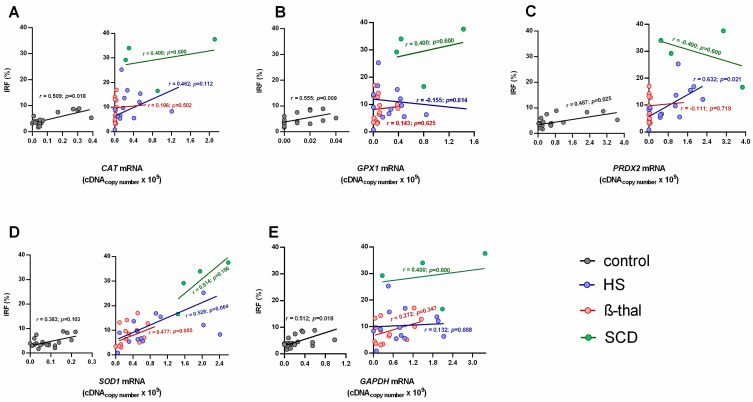
Percentage of immature reticulocyte fraction (IRF, %) versus mRNA levels of catalase (*CAT*, (**A**)), glutathione peroxidase 1 (*GPX1*, (**B**)), peroxiredoxin 2 (*PRDX2,* (**C**)), superoxide dismutase 1 (*SOD1*, (**D**)) and glyceraldehyde-3-phosphate dehydrogenase (*GAPDH*, (**E**)) for the control (*n* = 21), hereditary spherocytosis (*n* = 13), sickle cell disease (*n* = 4) and β-thalassemia (*n* = 14) groups. Spearman’s rank correlation coefficient was used to evaluate relationships between sets of data; *p* < 0.05 was considered statistically significant. β-thal, β-thalassemia; HS, hereditary spherocytosis; SCD, sickle cell disease.

When evaluating the relationships between reticulocyte maturity indices and the mRNA levels of the studied genes, we found that for the control group, the transcripts of all enzymes correlated negatively and significantly with the LFRs, and positively and significantly with the IRF, except for *SOD1* (Figure 2 and Figure 3).

In the NIHA groups, *PRDX2* mRNA levels were negatively correlated with LFRs and positively correlated with the IRF for HS patients (Figure 2 and Figure 3).

## 3. Discussion

Reticulocytes still have mRNA, providing a reservoir of information regarding their erythropoietic status. Intracellular RNA levels are directly correlated to fluorescence intensity and, thus, to the degree of reticulocyte maturation [4,5,9,10,11,12,13,14,15]. Several studies analyzed reticulocyte maturity indices in β-thal [6,8] and HS [7], reporting an increase in MFRs and HFRs and/or in IFRs, which indicated an increase in immature reticulocytes.

Herein, we studied the correlation between reticulocyte maturity indices for the first time, as given by automatic blood cell counters and the mRNA levels of antioxidant enzymes (*CAT, GPX1, PRDX2* and *SOD1*) in reticulocytes from healthy individuals and from patients with HS, β-thal or SCD.

When compared to the NIHA groups, reticulocytes from the control group showed the lowest mRNA content and, thus, were the more mature reticulocytes, as shown by the highest LFRs and the lowest IFRs (Table 1). Considering that the mRNA levels of the genes on study correspond to the copy number of RNA templates still untranslated during the final maturation stage of the reticulocyte in the bloodstream, the transcriptome observed in healthy individuals seems to follow the order *GPX1* < *GAPDH* < *SOD1* < *CAT* < *PRDX2* (Figure 1A–E). This is possibly in line with the relative amount of each enzyme that will be needed to support a normal RBC lifespan; actually, Prx2, the third most abundant protein in this cell [30], presents the highest transcript values. Our data support that as reticulocytes mature, the relative amounts of all RNA transcripts of these enzymes decrease as the proteins are synthesized, which is consistent with the decrease in immature circulating reticulocytes (IFRs) and the increase in late-stage circulating reticulocytes (LFRs) (Figure 2 and Figure 3).

Most of the correlations between the reticulocytes’ maturity indices and the mRNA levels for each enzyme were not statistically significant in HS, SCD and β-thal patients (Figure 2 and Figure 3). Thus, proportionality does not exist between the amount of total RNA and the levels of the enzyme transcripts in reticulocytes observed in controls. This might result from altered erythropoietic activity, which leads to changes in reticulocyte maturation and in their release from bone marrow, or from altered protein translation. An *et al.* [31] showed that the transcriptome composition during human terminal erythroid differentiation greatly changes from one stage to another. Given the different maturity indices presented by HS, SCD and β-thal patients, between each other and the controls (Table 1), it is reasonable that their transcriptome might reflect distinct temporal patterns of gene expression between more immature and more mature reticulocytes. However, it might also result from adaptive changes in erythroid cells due to alterations in erythroid cell components and/or in the erythropoietic environment.

Comparing the amounts of reticulocyte RNA, we found that for the three NIHA groups, the sequence order was *GAPDH* < *GPX1* < *SOD1* < *CAT* < *PRDX2*, which is different from that found for the control group (see above, Figure 1A–E). This change may reflect the differences in reticulocyte maturity, but it may also reflect different needs in the protein synthesis of the main redox enzymes. The quantity of *GAPDH* transcripts appears to be proportional to the reticulocyte maturity indices; however, for *CAT, GPX1, PRDX2* and *SOD1*, this proportionality is not evident (Table 1 and Figure 1E). In fact, HS and β-thal patients with similar IRF, LFRs and anemia severity (Table 1) presented clearly distinct quantities of mRNA transcripts (Figure 1A–D). This shows that reticulocytes with similar maturation development presented very different transcriptomes, which leads us to hypothesize that other factors than maturity may influence the mRNA levels of these redox enzymes. Probably, an adaptive response to an abnormal erythropoiesis and/or to an altered erythropoietic milieu occurs, such as in inflammatory or oxidative environments [3,32,33,34].

HS patients (all unsplenectomized) showed a significantly higher RPI and reticulocytosis compared to controls (Table 1). These common findings in HS patients [7,14,35] were associated to significantly lower LFRs and a higher IRF, evidencing the erythropoietic response to correct anemia by increasing RBC production and releasing more immature reticulocytes with significantly higher mRNA levels (Figure 1), likely due to incomplete/delayed protein synthesis. We must also consider that this increase in untranslated transcripts may result from their accumulation due to an impairment in protein synthesis, or from a higher stimulus for transcription of antioxidant enzymes in order to face an erythroid oxidant environment. Interestingly, in HS patients, *PRDX2* showed a strikingly increased mRNA value, as compared to the other antioxidant enzymes, accompanied by significant correlations with LFRs and the IRF (Figure 2 and Figure 3). This supports that Prx2 has a main role in the regulation of the redox metabolism of RBCs in HS patients, as reported elsewhere [36,37], possibly intervening as early as the erythropoietic development.

Patients with SCD presented a similar number of reticulocytes to HS patients but showed more severe anemia (Table 1). Their RPI values were significantly lower than in HS, showing that in SCD, the erythropoietic response is less efficient or impaired. In fact, we observed the most immature reticulocyte profile in SCD, with the highest IRF and the lowest LFRs (Table 1), as reported by others [14,15,38,39,40]. This was also supported by the extremely high levels of all mRNA transcripts, especially when comparing their average increase to the controls (Figure 1F). It was shown that stress erythropoiesis occurs in SCD patients [3,41], with a lower number of erythropoietic cells within a hypoxic and inflammatory environment [42]. This unique milieu in the bone marrow and in the peripheral circulation might certainly underlie the more severe anemia and the highest reticulocyte immaturity, as compared to the HS and β-thal groups; it may also explain the lower RPI (Table 1).

In β-thal patients, in spite of normal reticulocyte counts, the IRF was higher and LFRs lower than in controls (Table 1), showing a premature reticulocyte release, as reported by others [6,7,8,14,15,36,43]; this was associated with increased *GPX1* and *SOD1* mRNA levels (although not as high as in SCD and HS) (Figure 1B,D). Romanello *et al.* [29] also reported significantly increased transcripts of *GPX1* and *SOD1* genes in β-thal intermedia patients. These authors [29] also reported an increase in *CAT* mRNA levels and decreased *PRDX2* levels, while our data only indicate trends, which is possibly explained by the less severe form of β-thal (minor trait) of our patients.

It is important to notice that β-thal had a similar reticulocyte maturity profile to HS, but the RPI was significantly lower (Table 1), denoting a noteworthy ineffective erythropoiesis [44,45,46]. The altered erythropoiesis may explain the observed similarity in circulating reticulocyte maturity between HS and β-thal patients, which was simultaneous with very different pattern in mRNA relative levels of the antioxidant enzymes (Figure 1F). The β-thal patients showed the lowest mRNA levels among the pathologic groups (Figure 1A–E), presenting *CAT, PRX2* and *GAPDH* mRNA levels similar to those of the control group. In β-thal, erythropoiesis is characterized by an early-stage enhanced erythroblast proliferation, in parallel with limited erythroid differentiation and increased apoptosis during the late stages of erythropoietic development [44,45,46]. It appears that when the erythroblasts are able to differentiate, they give rise to reticulocytes which present an mRNA profile closer to that of healthy individuals than to those of HS or SCD patients. Unlike β-thal, the latter two present the typical hallmarks of stressed erythropoiesis associated with inflammation [41,42,47,48]; thus, it is possible that these very different pathologic erythropoietic developments might result in the transcriptome-distinct patterns that we describe herein.

In conclusion, similar reticulocyte maturity indices, as observed in β-thal and HS, were linked to completely different mRNA level profiles; on the contrary, SCD and HS cases with distinct reticulocyte immaturity showed similar patterns of transcripts. Our results suggest that these differences in the relative mRNA levels for the studied enzymes are not entirely related to the maturity of reticulocytes but appear to also reflect the response to changes within the erythroid cells and/or in the bone marrow environment (oxidative, inflammatory) characteristic of each anemia. That is, the reticulocytes released into the bloodstream already present adaptive modifications according to each NIHA to assure the development of mature RBCs equipped with the needed antioxidant potential to achieve a lifespan as close as possible to normal.

## 4. Materials and Methods

### 4.1. Subjects

This study was conducted in accordance with the Declaration of Helsinki and approved by the Ethic Committees of Centro Hospitalar e Universitário de São João, Porto, and Centro Hospitalar Universitário de Santo António, Porto. All participants, or their legal representatives, gave their informed consent to participate in the study. Patient selection was performed by clinical hematologists according to the study’s parameters.

We studied 81 individuals, including 31 healthy individuals (control group) and 50 subjects diagnosed with NIHA: 22 HS patients (all unsplenectomized), 6 SCD patients ( homozygous SCD (S/S)and 20 β-thal patients (all minor trait). Blood samples were collected (5 mL, using EDTA as anticoagulant) during the routine follow-up of the patients and when in steady-state pathological conditions. The patients had not received blood transfusions in the 4 months prior to this study.

No statistically significant (χ^2^) differences were found for sex (male/female: 52/48%, 52/48%, 25/75%, 50/50%) and age (27 ± 2, 24 ± 5, 15 ± 5, 32 ± 6 years) between control, HS, SCD and β-thal groups, respectively.

### 4.2. Evaluation of Hematological Parameters

Complete blood count, reticulocyte count and reticulocyte maturation indices, namely, low-fluorescence reticulocytes (LFRs), medium-fluorescence reticulocytes (MFRs), high-fluorescence reticulocytes (HFRs) and immature reticulocyte fraction (IRF, sum of MFRs plus HFRs) [10,49], were also evaluated (Sysmex XN-9000^®^). The reticulocyte production index (RPI) was calculated according to Hillman [50].

Due to COVID-19 hospital constraints during the time period of sample collection (2019–2022), the reticulocyte maturation indices were only determined for 21 controls, 13 HS patients, 4 SCD patients and 14 β-thal patients. Nonetheless, after performing statistical analysis utilizing both the larger or smaller sample number of individuals, we found that the results (hematological and reticulocyte parameters and reticulocyte mRNA levels) were similar, independently of case number, when comparing the different groups (Table S1 of Supplementary Data).

### 4.3. Separation of Reticulocytes

After a double density gradient (Histopaque 1.077 and 1.119, Sigma-Aldrich, St. Louis, MO, EUA) centrifugation (700× *g*, 25 °C, 30 min) of whole-blood samples (500.0 μL), plasma and leukocytes were discarded, and RBCs were isolated and washed two times with phosphate-buffered saline (PBS) solution (pH 7.4). Then, reticulocytes were separated from mature RBCs using the manual column-based magnetic cell isolation—MACS^®^—separation technology (MidiMACSTM kit equipped with LS columns, Miltenyi Biotec, Bergisch Gladbach, Germany) and the MACS^®^ MicroBeads (human CD71 MicroBeads, Miltenyi Biotec), according to the manufacturer’s instructions.

Briefly, a suspension containing 1.8 × 10^9^ of erythrocytes was prepared in MACS^®^ buffer (PBS pH 7.2, 0.5% bovine serum albumin and 2.0 mM EDTA, sterilized and degassed). Upon centrifugation (300× *g*, 4 °C, 10 min), the pellet was resuspended in 120.0 μL of buffer, and 80.0 μL of CD71 MicroBeads was added for a 15 min incubation at 4 °C. After washing, the reticulocytes were separated using the MidiMACS^TM^ separator. The magnetically labeled CD71+ cells (reticulocytes) were retained on the column and later eluted with 6 mL of buffer. 

The obtained cell suspension was centrifuged (1000× *g*, 4 °C, 5 min) and TRI reagent solution (Sigma) was added to the final pellet (reticulocytes) for cell lysis and conservation; the samples were stored at −80 °C until RNA extraction. 

### 4.4. qRT-PCR Analysis

Phenol-chloroform RNA extraction from reticulocytes was performed according to the manufacturer’s instructions (TRI Reagent^®^ Protocol, Sigma-Aldrich, St. Louis, MO, EUA). Briefly, after thawing, chloroform was added to the samples that were prepared as described in Section 2.3, and after centrifugation (12,000× *g*, 15 min, 4 °C), RNA was precipitated with isopropanol from the aqueous phase. Samples were again centrifuged (12,000× *g*, 10 min, 4 °C), and the RNA pellet was resuspended and washed in 75% ethanol (7500× *g*, 5 min, 4 °C). Finally, the RNA pellet was dissolved in RNase-free water and stored at −80 °C until further assayed.

RNA concentrations were measured by NanoDrop (ND-1000 Spectrophotometer, NanoDrop Technologies Inc., Wilmington, DE, EUA). cDNA was obtained by reverse transcription of RNA templates using the Xpert cDNA synthesis kit (GRiSP, Porto, Portugal), following the manufacturer’s instructions.

For amplification in qPCR reactions (Applied Biosystems StepOnePlus Real-Time PCR system, Thermo Fisher Scientific, Waltham, MA, EUA), Xpert Fast SYBR 2X mastermix (GRiSP), custom primers (Table 2) and 1 ng of cDNA were used. The cDNA levels were assessed by performing a calibration against a standard curve of known amounts of synthesized cDNA. To establish the standard curve for each gene, a cDNA stock solution in the range of 10^2^ to 10^−4^ ng of nucleic acid/reaction was used (Figure S1 of Supplementary Data). The nucleic acid amount in each sample was expressed in copy numbers of cDNA according to Dorak [51].

Alongside with the antioxidant enzyme genes (*CAT*, *GPX1*, *PRDX2* and *SOD1*), *GAPDH* was also evaluated because glyceraldehyde-3-phosphate dehydrogenase (G3PD) synthesis still occurs at the reticulocyte stage [52], and this gene has been extensively used in gene expression studies, due to its ubiquitous existence. Moreover, G3PD linkage at the erythrocyte membrane has been proposed as a marker of membrane destabilization and OS [36,53], and it was found to be correlated to reticulocyte count in HS patients [54].

To compare the relative amount of RNA transcript levels between each NIHA and the control group, the ratios between the mean copy number of cDNA value for each NIHA/control pair were calculated.

### 4.5. Statistical Analysis

IBM SPSS Statistics 29 for Windows (SPSS Inc., Chicago, IL, EUA) was used. Data normality was assessed by the Shapiro–Wilk test. Due to their non-Gaussian distribution, data are presented as median values (interquartile range). Group differences were examined via the Pearson Chi-Square and Kruskal–Wallis H tests, and upon statistical significance, pairwise comparisons were made using the Mann–Whitney U test. Associations between data were assessed by Spearman’s rank correlation coefficient. Statistical significance was reached when *p* < 0.05.

## Figures and Tables

**Table 1:** Hematological data, reticulocyte count, reticulocyte production index, reticulocyte maturity indices and immature reticulocyte fraction for control, hereditary spherocytosis, sickle cell disease and β-thalassemia groups.

	Control (*n* = 31)	HS (*n* = 22)	SCD (*n* = 6)	β-Thal (*n* = 20)
RBCs (×10^12^/L)	4.9 (4.5–5.1)	4.0 (3.4–4.4) *	2.6 (2.2–3.5) *^a^	5.7 (5.1–5.9) *^ab^
Hb (g/L)	147 (137–154)	123 (113–131) *	81 (76–92) *^a^	112 (104–126) *^b^
MCV (fL)	88 (87–90)	86 (82–92)	86 (79–99)	63 (60–70) *^ab^
MCH (pg)	30 (29–31)	30 (29–33)	30 (26–35)	20 (19–22) *^ab^
RDW (%)	12.7 (12.4–13.1)	16.8 (14.2–18.6) *	19.7 (18.5–21.1) *^a^	15.9 (15.3–16.6) *^b^
RET (×10^9^/L)	56 (49–71)	215 (143–263) *	177 (138–196) *	77 (52–107) *^ab^
RET (%)	1.2 (1.0–1.4)	5.4 (3.8–6.8) *	5.8 (4.8–7.3) *	1.5 (1.0–1.9) ^ab^
RPI	1.07 (0.86–1.40)	2.41 (2.03–2.82) *	1.84 (1.39–2.55) *	0.67 (0.53–0.81) *^ab^
	Control (*n* = 21)	HS (*n* = 13)	SCD (*n* = 4)	β-thal (*n* = 14)
LFRs (%)	96.1 (94.3–96.8)	91.0 (85.4–94.1) *	68.4 (63.3–80.2) *^a^	92.2 (87.1–95.9) *^b^
MFRs (%)	3.9 (3.9–5.6)	8.0 (5.4–10.8) *	18.9 (12.5–19.7) *^a^	7.2 (3.4–11.3) *^b^
HFRs (%)	0.2 (0.0–0.4)	0.9 (0.6–3.8) *	12.8 (6.8–17.4) *^a^	0.9 (0.3–1.7) *^b^
IRF (%)	3.9 (3.2–5.6)	9.0 (6.0–14.6) *	36.7 (19.8–36.7) *^a^	7.8 (4.1–12.9) *^b^

Data are presented as median (interquartile range). Mann–Whitney U test was used to compare differences between groups; *p* < 0.05 was considered statistically significant. * *p* < 0.05 vs. control group; ^a^ *p* < 0.05 vs. HS patients; ^b^ *p* < 0.05 vs. SCD patients. β-thal, β-thalassemia; Hb, hemoglobin; HFRs, high-fluorescence reticulocytes; HS, hereditary spherocytosis; IRF, immature reticulocyte fraction; LFRs, low-fluorescence reticulocytes; MCH, mean corpuscular hemoglobin; MCV, mean corpuscular volume; MFRs, medium-fluorescence reticulocytes; RBCs, red blood cells; RDW, red cell distribution width; RET, reticulocytes; RPI, reticulocyte production index; SCD, sickle cell disease.

**Table 2:** List of primer sequences, annealing temperatures and reaction concentrations.

Gene		Primer Sequences (5′→3′)	T_a_ (°C)	Concentration (nM)
*CAT*	F	ACT GTT GCT GGA GAA TCG GG	60	250
	R	TCC CTG ATG AAG AAA ATG GGG G		
*GPX1*	F	GGT CTG GTC TTC AGC TAC CC	60	250
	R	ACC AGT TTC TTC CGG ATG GC		
*PRDX2*	F	CCT GAA CAT CCC CCT GCT TG	60	250
	R	AGT GAT CTG GCG AAG GAC AC		
*SOD1*	F	GAG AGG CAT GTT GGA GAC TT	60	200
	R	TCT GCT TTT TCA TGG ACC ACC		
*GAPDH*	F	TAT GAC AAC AGC CTC AAG AT	60	200
	R	GAG TCC TTC CAC GAT ACC		

*CAT*, catalase; F, forward; GAPDH, glyceraldehyde-3-phosphate dehydrogenase; *GPX1*, glutathione peroxidase 1; *PRDX2*, peroxiredoxin 2; R, reverse; *SOD1*, superoxide dismutase 1; Ta, annealing temperature.

## Data Availability

The raw data supporting the conclusions of this article will be made available by the corresponding authors on request. The data are not publicly available due to privacy and ethical reasons.

## Supplementary Materials

The following supporting information can be downloaded at https://www.mdpi.com/article/10.3390/ijms25042159/s1.
