# Supplementary material for: Reticulocyte Antioxidant Enzymes mRNA Levels versus Reticulocyte Maturity Indices in Hereditary Spherocytosis, β-Thalassemia and Sickle Cell Disease
Source: Int J Mol Sci. 2024 Feb 10;25(4):2159. doi: 10.3390/ijms25042159 (PMC10889157; doi:10.3390/ijms25042159)
Supplement: Supplementary file 1 [file ijms-25-02159-s001.zip › ijms-2849786-supplementary.pdf]

Table S1. Hematological data, reticulocyte count, reticulocyte production index and reticulocyte's mRNA levels of catalase, glutathione peroxidase 1, peroxiredoxin 2, superoxide dismutase 1 and glyceraldehyde-3-phosphate dehydrogenase for control, hereditary spherocytosis, sickle cell disease and β-thalassemia groups.

	**Control (*n* = 21)**	**HS (*n* = 13)**	**SCD (*n* = 4)**	**β-thal (*n* = 14)**
RBC ($x10^{12}$/L)	4.83 [4.52-5.16]	4.00 [3.40-4.50]*	2.60 [2.22-3.28]*[a]	5.27 [5.02-6.08]*[ab]
Hb (g/L)	146 [136-152]	123 [108-131]*	82 [76-91]*[a]	113 [103-128]*[b]
MCV (fL)	88.4 [86.2-91.2]	84.3 [81.2-91.8]	85.5 [79.2-105.7]	62.6 [60.2-70.9]*[ab]
MCH (pg)	30.2 [28.9-31.0]	29.5 [28.7-32.6]	30.1 [26.6-38.3]	20.0 [19.3-22.4]*[ab]
RDW (%)	12.7 [12.4-13.3]	18.1 [14.2-19.9]*	19.4 [18.4-20.8]*	16.1 [15.3-17.2]*[b]
RET ($x10^{9}$/L)	60 [52-75]	214 [87-244]*	140 [138-186]*	84 [52-112][ab]
RET (%)	1.24 [1.04-1.48]	5.65 [2.52-6.82]*	4.80 [4.71-6.00]*	1.56 [1.00-1.90][ab]
RPI	1.12 [0.80-1.49]	2.41 [1.84-3.06]*	1.84 [1.34-3.41]*	0.74 [0.50-0.84]*[ab]
CAT mRNA (cDNA $_{copy\ number}$ x 10^{6})	5.0 [3.5-12.0]	177.0 [88.5-464.0]*	604.5 [249.0-1810.8]*	12.5 [4.00-26.5][ab]
GPX1 mRNA (cDNA $_{copy\ number}$ x 10^{6})	0.0 [0.0-0.20]	2.50 [0.35-4.40]*	6.20 [3.88-12.72]*	0.70 [0.18-1.00]*[b]
PRDX2 mRNA (cDNA $_{copy\ number}$ x 10^{6})	21.0 [11.5-73.0]	1090.0 [258.0-1495.0]*	2000.0 [602.8-3673.0]*	20.0 [3.0-43.0][ab]
SOD1 mRNA (cDNA $_{copy\ number}$ x 10^{6})	0.60 [0.25-1.30]	5.90 [3.65-15.35]*	17.60 [14.72-24.38]*	2.50 [1.12-5.20]*[ab]
GAPDH mRNA (cDNA $_{copy\ number}$ x 10^{6})	0.12 [0.08-0.35]	0.80 [0.33-1.44]*	1.79 [0.58-3.06]*	0.26 [0.07-1.06]

Data are presented as median (interquartile range). Mann-Whitney U test was used to compare differences between groups; $p < 0.05$ was considered statistically significant. *$p < 0.05$ *vs.* control group; [a]$p < 0.05$ *vs.* HS patients; [b]$p < 0.05$ *vs.* SCD patients. β-thal, β-thalassemia; *CAT*, catalase; *GAPDH,* glyceraldehyde 3-phospate dehydrogenase; *GPX1*, glutathione peroxidase; Hb, hemoglobin; HS, hereditary spherocytosis; MCH, mean corpuscular hemoglobin; MCV, mean cell volume; *PRDX2*, peroxiredoxin 2; RBC, red blood cells; RDW, red cell distribution width; RET, reticulocytes; RPI, reticulocyte production index; SCD, sickle cell disease; *SOD1*, superoxide dismutase
